# Supplementary material for: Gas1 regulates embryonic tongue muscle proliferation, differentiation and maturation via alternative pathways to Hedgehog signaling
Source: Development. 2025 Oct 10;152(19):dev204868. doi: 10.1242/dev.204868 (PMC12539211; doi:10.1242/dev.204868)
Supplement: Supplementary information [file develop-152-204868-s1.pdf]

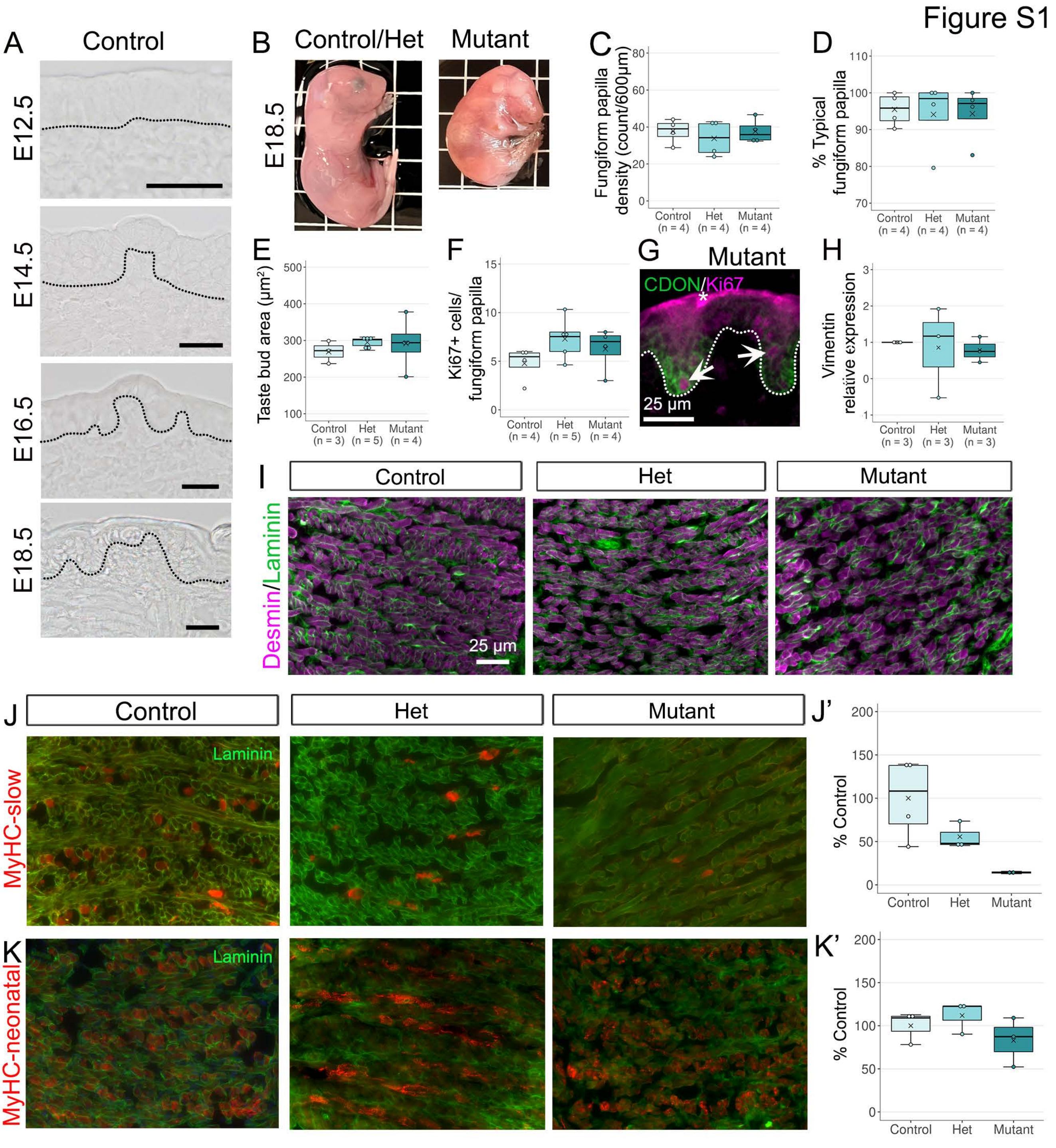

Figure S1. Craniofacial abnormalities and tongue analyses after *Gas1* global deletion. A. X-gal staining of sagittal tongue sections at E12.5, E14.5, E16.5, and E18.5 in *lacZ*-negative $Gas1^{-/-}$ tongues. **B**. At E18.5, embryo size is substantially decreased in Mutant ($Gas1^{lacZ/lacZ}$) compared to Control ($Gas1^{+/+}$) or Het ($Gas1^{lacZ/+}$). **C-F**. At E18.5, fungiform papilla density (C), morphology (D), taste bud area (E), and proliferating cells (F) are similar between Control, Het,

Development • Supplementary information

and Mutant. **G**. Immunostaining with CDON (green) and Ki67 (purple) in Mutant to demonstrate co-expression (arrows) in cells where K5 is eliminated (Figure 3I'). **H**. qPCR analysis indicates similar vimentin gene expression in Control, Het, and Mutant groups at E18.5. **I**. Immunostaining of muscle fibers with desmin (purple) and laminin (green) demonstrates intact muscle fusion in Control, Het, and Mutant groups. **J-K'**. Immunostaining of muscle fibers with laminin (green) and Myosin Heavy Chain (MyHC)- slow (J) and -neonatal (K). MyHCslow and MyHC-neonatal fibers are quantified and presented as % Control. MyHC-slow fibers are substantial decreased in
Mutant as compared to Control and Het (J'). MyHC-neonatal fibers are similar in all groups (K'). Scale bar in I applies to J,K. Whiskers represent median ± maximum/minimum. X denotes
mean. (one-way ANOVA with Tukey's HSD posthoc test). Number of tongues (n) analyzed is 3 unless noted otherwise in parentheses. Scale bars are 25 μm in A. Whiskers represent median +/- the maximum or minimum (one-way ANOVA).

Figure S2

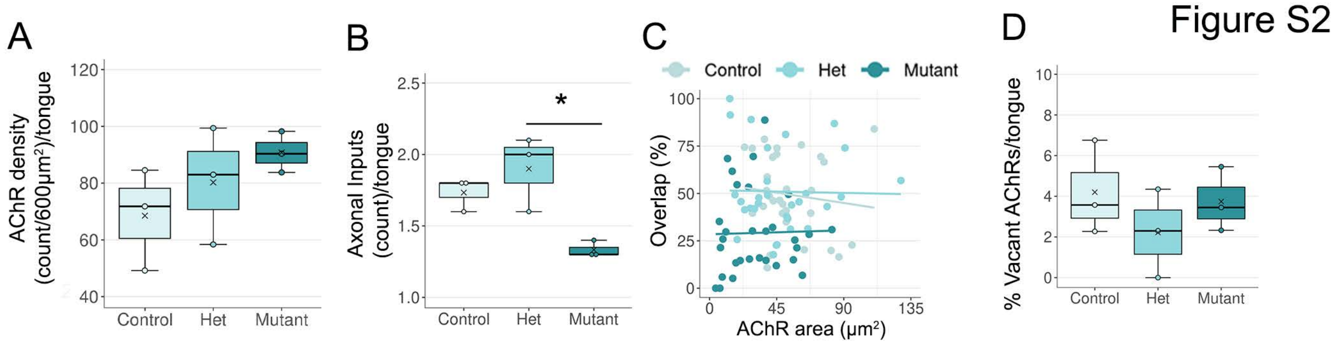

Fig. S2. A. AChR density demonstrates an increasing trend in Mutant compared with Control group. **B**. The number of axonal inputs to Mutant AChR is significantly reduced compared to Het. **C**. SNAP25 + presynaptic fibers overlap with individual AChRs and area occupied by AChRs demonstrate no correlation in all groups. **D**. Vacant AChRs, demonstrating no overlap between SNAP 25 and α-bungarotoxin, were unchanged in Control and Het groups compared with Mutant. Whiskers represent median +/- the maximum or minimum. *$p \leq 0.05$ (two-way ANOVA, C or one-way ANOVA, A,B,D). Number of tongues analyzed is 3 in all groups.

Table S1. Description of primary and secondary antibodies used in this study.

Antibody	Dilutions	Source	Cat. #	Lot #
**Primary Antibodies**				
Chicken anti-vimentin	1:2000	Millipore	AB5733	3990225
Goat anti-BOC	1:100	R&D Systems	AF2385	UKN012302A
Goat anti-CDON	1:100	R&D Systems	AF2429	ULP0122021
Goat anti-CDON	1:100	Invitrogen	PA5-47315	ZI4477126
Goat anti-Ecad	1:1000	R&D Systems	AF748	CYG0419101
Goat anti-GAS1	1:100	R&D Systems	AF2644	VFH0120041
Goat anti-Shh	1:500	R&D Systems	AF445	BIQ0423081
Mouse anti-Pax7	1:1000	DSHB	PAX7-s	10/14/21
Mouse anti-MyHC-embryonic	1:25	DSHB	F1.652	2/20/25
Mouse anti-MyHC-neonatal	1:25	DSHB	N3.36	9/7/23
Mouse anti-MyHC-slow	1:25	DSHB	BA-D5	8/17/23
Rabbit anti-desmin	1:200	Invitrogen	PA1-37556	YD3871962B
Rabbit anti-GFP	1:1000	Abcam	Ab6556	GR3404234-1
Rabbit anti-K5	1:500	Abcam	EP1601Y	GR3292032-2
Rabbit anti-Ki67	1:1000	Abcam	AB16667	GR3313195-53
Rabbit anti-myogenin	1:500	Invitrogen	PA5-119967	YC3875858C
Rabbit anti-MyHC	1:250	Invitrogen	MA5-32555	YC3871824A
Rabbit anti-NF	1:1000	Novus Bio	NB300-135	216-070620
Rabbit anti-P2X3	1:1000	Novus Bio	NBP2-33848	A106775
Rabbit anti-RFP	1:1000	Rockland	600-401-379	42896
Rabbit anti-SNAP25	1:1000	Invitrogen	PA5-87549	ZC4250814B
Rabbit anti-β-tubulin	1:1000	Abclonal	AC008	3507438004
Rat anti-K8	1:1000	DSHB	TROMA-I	12/12/19
Rat anti-laminin	1:1000	Invitrogen	MA1-06100	YI4039621
α-bungarotoxin (BTX)	1:100	ThermoFisher	T1175	159542
**Secondary Antibodies**				
Anti-rabbit Alexa Fluor 488	1:500	JIR*	711-545-152	159923
Anti-rabbit Rhodamine	1:500	JIR	711-025-152	160846
Anti-rabbit Cy5 650	1:500	JIR	711-175-152	160141
Anti-mouse Alexa Fluor 488	1:500	Invitrogen	A21202	2147618
Anti-mouse Alexa Fluor 568	1:500	Invitrogen	A10037	2110843
Anti-mouse (IgM) Cy3	1:500	JIR	715165020	168771
Anti-goat Alexa Fluor 488	1:500	JIR	705-545-147	155707
Anti-goat Rhodamine	1:500	JIR	705-025-147	153624
Anti-rat Alexa Fluor 488	1:500	JIR	712-545-153	159115
Anti-rat Rhodamine	1:500	JIR	712-025-153	159229
Anti-chicken Rhodamine	1:500	JIR	703-025-155	157517

*Jackson ImmunoResearch
